# Construction and Application of Dynamic Evaluation System and Educational Model of College Students' Mental Health

**DOI:** 10.3389/fpubh.2022.888052

**Published:** 2022-04-29

**Authors:** Yaling Li

**Affiliations:** Department of Education, Shenzhen Technology University, Shenzhen, China

**Keywords:** dynamic evaluation model, block chain, education model, mental health, self-awareness, management

## Abstract

The aim of the paper is a dynamic evaluation system that provides support to the students' mental health. Mental health contains students' feelings, psychological, and social well-being. Extracurricular activities, professional and innovative skills, self-awareness, emotional management, cultural norms, and personality development are the essential factors in a dynamic evaluation system for students' mental health. Extracurricular activities support learning additional things except for the subjects. Thinking skill is being an optimistic idea to mental health. Through professional and innovative skills, students can express their thoughts and ideas with others. Understanding one's feelings are considered self-awareness. It supports finding the good and uniqueness of a particular person. Emotional management accepts and controls the feelings of the students. Virtue, ethics, honesty, loyalty, responsibility, positive thoughts, relational skills are the important factors in cultural norms. Feelings and ways of behavior affect mental health in personality development. Self-confidence is the main element in personality development. This article engaged to adopt a descriptive research method to present a perfect model for this research. The probability method (random sampling method) and non-probability method (purposive sampling method) were utilized for this research. A total of 349 sample sizes of college students participated in the questionnaire survey and 12 sample sizes of college instructors responded to the face-to-face interview from the priority areas of the university. Finally, Thus, in this research, extracurricular activities and professional and innovative skills are dominant factors when examining other factors. These leading factors are expressed clearly by students to protect their mental health of the students.

## Introduction

Mental Health (MH) education has become an integral part of moral education in schools, and the study of student's psychology is an important task for educators (1). Dynamic evaluation is a type of interactive evaluation used in education. Dynamic evaluation is an outcome of the research examined by developmental psychologist Lev Vygotsky (2). Dynamic evaluation (DA) is a technique of organizing assessment that searches to identify skills or mental ability that a specific student obtains conjointly student's learning promising. The dynamic evaluation process gives priority to the educating procedure and nature of examiner investment. It is mainly shared and technique-based (3). The dynamic evaluation procedure and techniques should also be easy and realistic. The psychological quality of college students and the construction model of the analytical hierarchical procedure, the analytic hierarchical procedure, the basic procedure of psychological evaluation of college students, can be obtained (1). The benefit of dynamic evaluation is that it does not ignore anything in evaluation models, but use the elements they include from time to time. On top of that, on another side, dynamic access is not oriented on any of the familiar evaluation models (4). The goals of dynamic evaluation are to evaluate learning capability, to recognize learning procedures, and to educate the exclusive theorize strategies for problem-solving remain its major strength (5). No other evaluation tradition is so ambitious. The MH of college students is an important issue of current social responsibility and harmonized to manage the psychological circumstances of college students (6). Through music courses that colleges and universities hold themselves, the teacher reads a lot of literature and conducts fieldwork to present the full play for the potential functioning of creative intervention in the MH of college students (7).

Previous research shows that the needs of college students' health continue. Change increases mental health problems. The number of students entering college counseling centers has increased from high (8). Information requirements, when it comes to the most critical psychological issues, traditional psychological evaluation trains used to focus on the availability or absence of a psychiatrist, with a diagnosis associated with the observable sign of a problem and related conditions (9). With the very quick growth of the socio-economy, the life of the people is universal, but what to focus on psychological issues are creating an important issue that affects the safety of people's lives (10). Powerful efforts are a necessity for developing the mental health of college students (11). According to Kadison and Digeronimo, They discussed that there is a problem in mental health in college students. To encourage advice, research on mental health and treatment to mental health, the American psychiatric association builds up a task force on colleges (12). A problem in the United States, with a national community health research newsgathering that overall half the college pupils scrutinized met the test for a psychiatric disorder in the last year although <25% required treatment, and that the ratio for college students was not considerably dissimilar from non-students (13).

College students cannot able to find their mental health (MH) issues like physical problems. Some people are not ready to get treatment for their mental state, which quality affects their life in a lot of ways. The researcher finds that the person who has negative thoughts, pessimistic has low immunity power (1). Prayer and meditation help college students to present a quiet mind and feel calm. Students have to set realistic and high aims. It should express self-worth (14). It helps to avoid students' mental problems. Nowadays, early warning of psychological crisis is still traditional in most universities and the effect of early warning is minimal (1). The evaluation of the mental education of new-age college students is a large but closely joined fullness, and when analyzing the appropriate content, we must pay concentration to the fullness and systematic nature of the evaluation (15, 42–44). The goal of this research is through the dynamic evaluation system, can improve the mental ability of college students. The rest of this paper is structured as follows: In the Section Literature survey, a literature survey is given. Theoretical framework and hypothesis development are provided in the Sections Theoretical Framework and Hypothesis Development. The methodology can be seen in the Section Methodology. Results and discussion are presented in the Sections Result and Discussion. Finally, the Section Conclusion gives the conclusion.

### Literature Survey

Wang and Park (11) designed a management system of intelligent sports that is based on the technology of deep learning to solve the problems of college students' poor physical fitness and low level of efficiency in the management of sports venues. The age of the user, the status of physical health, Body mass index, and recommended sports programs suitable for students, were analyzed using an artificial intelligence recommendation algorithm. It was found that the teaching needs of colleges were met by the sports training environment instructional system and it promoted psychological education. Meng et al. (16) proposed a three-channel multi-feature fusion network based on the technology of Neural Network (NN), for predicting and identifying the MH problems of college students in the self- entrepreneurship environment. Inputs of the three-channel network are behavior features, visual features, and social relations. The results showed that the method has a possibility for the assessment of students' MH and solving MH problems of students.

Li and Yu (17) proposed a model of novel dual-branch [neural network for prediction of MH and computer vision-based facial emotion recognition] neural network for assessing the MH of college students. The paper proposed to combine the neural networks with Bayesian methods. The results proved the effectiveness and superiority of the model through the experiments of comparative and simulations. Wang and Wang (18) constructed a comprehensive evaluation system by using the Minnesota personality questionnaire (MMPI), the personality questionnaire (EPQ), and the depression experience questionnaire (DEQ) to evaluate the mental health of clinicians. Factor analysis was used to evaluate mental health from various factors that reflected the status of the mental health of clinicians. The results proved that the evaluation system has good reliability and credibility and the status of clinicians' mental health can be classified.

Jing (19) proposed a study on the assessment of MH intelligence based on multi-score information fusion. Based on the Circos diagram multisource information fusion visualization was designed by UPI and SCL-90 data and task requirement analysis. The parallel coordinate visualization was established. A total of 278 university students UPI and SCL-90 questionnaire data's are used for this study. The study analyzed the mental health problem of students. Yannam et al. (20) designed and developed a Privacy-Preserving Automated Stress Monitoring System to monitor mental health issues. Data collection was done by using an android application that was built for the study and machine learning-driven insights on stress conditions of individuals were provided. The predictions on the data collected were validated by using Perceived Stress Scale (PSS) and Daily Stress Inventory (DSI). Zhang (21) studied and analyzed the common problems such as lack of teachers, imperfect systems, etc., in the mental health education of college students and presented a few countermeasures for optimizing the mental health education of college students. The study identified that still there were problem that needs to further strengthen the measures of mental health for students.

## Theoretical Framework

**Figure 1:**
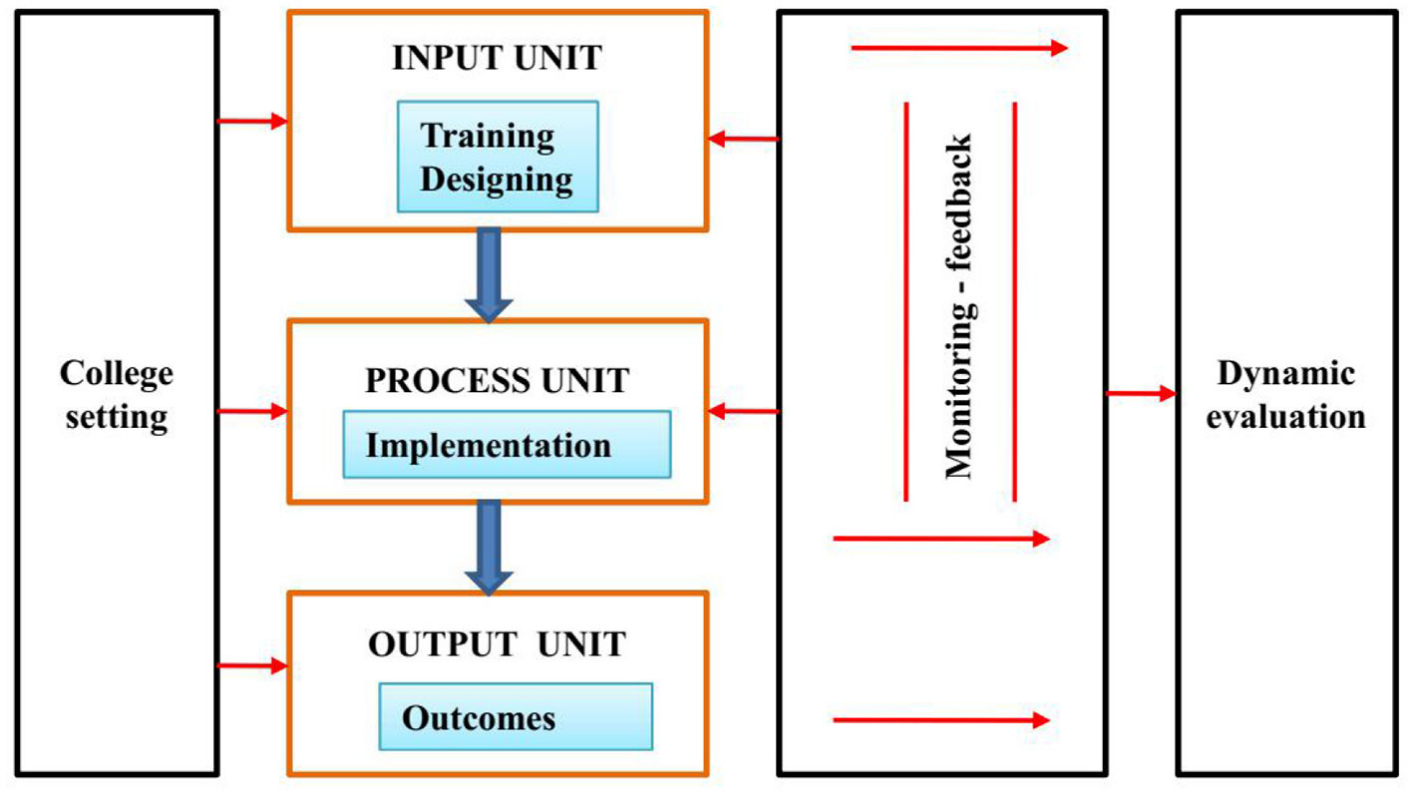
Dynamic evaluation model.

Dimitropoulos proposed a dynamic evaluation method in 1999. Figure 1 represents the Dynamic Evaluation model. Dimitropoulos is not on any basis known evaluation models because its structure is typically by an effortless set of various methods depending on the chance. In detail, based on the outlook of the dynamic evaluation method about “selection” and “dynamism” (4). The participatory model was also used to access the mental health of the students in past research. A participatory intervention model is ideal is distinguished by a combined process that partners create make together interventions to assist the progress of individual and educational change. Participatory activity research expresses a foundation for the idea of developing mental health programs in colleges and communities. The participatory model is an intense educating method for performance that enlists the inherent and particular intelligence of shareholders to build formalized and mutual representations of reality (22).

In the course of mental health education, colleges and universities should be enthusiastic to gather psychiatrists to execute focus theoretical teaching according to the various developmental steps of the students. Theoretical teaching content should express the elements of higher education such as health professional personality education, post-contextual psychology adaptation education, mutual harmony education, psychology education after disappointment, and many more (21). Mental health is an important public health issue. Overall, regulators, mental health specialists, and instructors fulfill the necessities that colleges are allowed to play in identifying theMH essential of youngsters. Canadian mental health policies accepted the acts of intervening in school-based mental health. And Ontario's policy is considered as an evergreen framework for students MH. Institutions are the perfect place to teach mental health education. Because the youngest people mostly depend on institutions. And classroom-oriented education is well-known to the students. Including mental health education into students' curriculum may easily reach the students day by day. And it is a part of students' daily activities. Also, younger's mental health takes a major part in instructor's duty (23).

Three components are being a mental health curriculum. Firstly, to develop knowledge about the significance of sentimental well-being (24). Secondly, ignore the prohibition around mental health (25). At last, stimulate the students to know who is in suffering from mental health (26). Instructors have to be optimistic and have unconditional self-esteem for their students. If the instructors concentrate on both things, the instructor can value the students with or without excellence in evaluation. Adding extra syllables is not a good mental health curriculum. The lecturer has to teach the skills for mental health through textbooks and tests. An interactive session takes a major part in evaluation by counselors of every institution. Music therapy activities help the mental health development of college students. The college music therapy curriculum system is a friendly system to everyone like college students (27).

**Figure 2:**
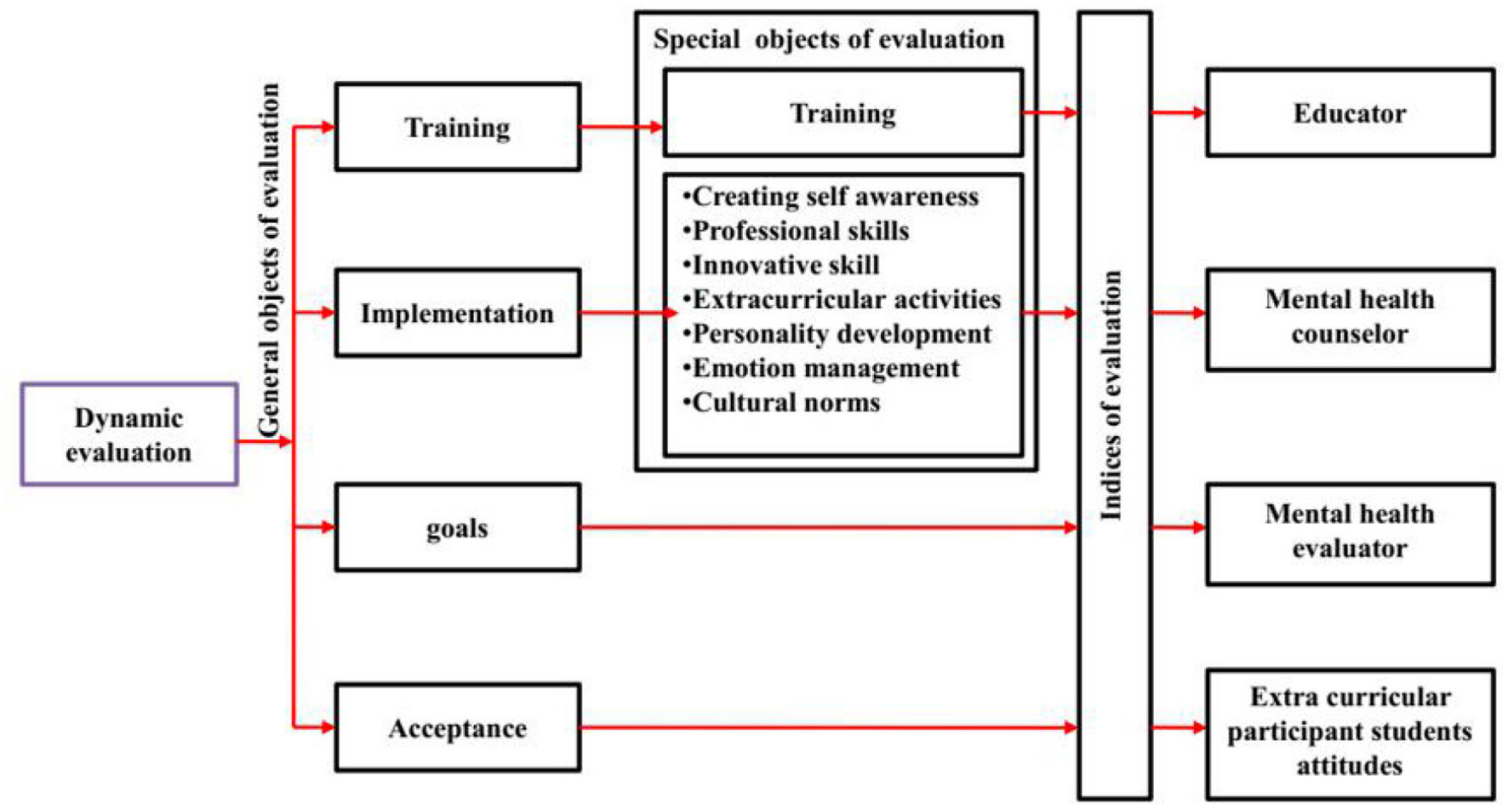
Dynamic evaluation of education model improving mental health.

In the traditional teaching method, instructor-centered is the major factor to affects students' mental health. Recently, a modern teaching method follows by colleges. Student-oriented, work-oriented, resource-situated, interactive in nature, integrative in nature, and peer cooperation are the typical features in modern teaching methods. Peer cooperation, student-oriented and interactive is an essential thing in both traditional and modern teaching methods, which is the perfect platform to ignore isolation. Through this ignorance of isolation, students can maintain flawless mental health (28). Extracurricular activities, professional and innovative skills, self-awareness, emotional management, cultural norms, and personality development are the valued factors in a dynamic evaluation system for students' mental health. Figure 2 represents the dynamic evaluation of education model improving mental health.

## Hypothesis Development

### Creating Self-Awareness

Self-awareness means a better understanding of our feelings and behavior. Being self-aware can help individuals to recognize their feelings, thoughts, etc, and allow having better control over the emotions (29). Emotional well-being is considered as the positive state of emotion, it involves balancing emotions. It helps the individuals to identify what is best in them and makes them feel positive and thus improves the ability for focused thinking. Stress can affect emotional well-being and there the mental health is affected (30). Coping with stress is another important factor for maintaining and balancing good mental health (31). The education model must create awareness about the importance of emotional well-being, coping effectively with stress, etc among students to improve their mental health.

### Improve Professional Skills and Innovative Skill

Improving professional skills involves improving communication skills, improving thinking skills, etc. Communication has a positive relationship with students' mental health (32); improving the skill of communication in students helps increase mental health. Communication is a way of expressing our thoughts. A student with better communication skills will easily interact with others about his thoughts and can able to maintain good relationships thus strengthening his mental health. Innovative skills involve creativity; creativity skills can reduce depression and stress which are the elements that affect mental health (33). The education model should include courses that help in improving the professional and innovative skills of students and thereby improve their mental health.

### Extracurricular Activities

Extracurricular activities are activities performed by students which are not part of their normal curriculum. These activities help the students to learn new things. Participation in extracurricular activities has a major impact on the positive development of youth. These activities are considered as an asset that contributes to the well-being of individuals. Past studies suggest that joining extracurricular activities positively affects the improvement of mental health (34). Few studies have examined the relationship between mental health and participation in extracurricular activities. Active engagement in extracurricular activities will contribute to positive mental health. But spending more time in unstructured activities can negatively affect psychology (35).

### Personality Development

The personality of an individual describes the way of behavior, feelings, attitudes, and thoughts and influences mental health. The presence of personality disorder may negatively affect the mental health of an individual. In the process of shaping personality, education plays a major role by providing knowledge and skills (36). Education offers many opportunities to develop self-confidence which is the key factor in personality development (37). Developing a good personality will improve mental health; it can be attained through a better education model. So an education model must be structured in such a way that it must promote personality development and thus increase mental health.

### Emotion Management

Emotion management is the process in which an individual controls his emotional states (38). It involves recognizing the emotions and learning control of emotions. One of the essential features of mental health is emotion regulation (39). Emotion management is connected with mental health. Mental health refers to how healthy is our mind in processing and understanding information. Emotion management involves handling our emotions. Strategies for managing emotions must be taught through the education to improve the mental health of students.

### Cultural Norms

Cultural norms provide help to the students for managing mental health in good condition. Especially in cultural norms, moral quality supports the students to lead a stress-less life. Braveness, future thoughts, positive thoughts, relational skills, work ethic, confidence are the elements of virtue. Honesty, integrity, impartiality, loyalty, responsibility are the main factors in ethics. These qualities avoid violence, suicide, and mental illness of the students. Virtuous and ethical, are the moral values in cultural norms (40). These qualities secured the students from mental health issues. Thus, Cultural norms present the guidance to lead a better life with good mental health.

## Methodology

### Research Design

This paper intended to adopt a descriptive research design to provide a suitable model for a study. The main objective is to study and develop a dynamic evaluation system that manages the study programs and plans within the university to improve the mental health of college students. Both qualitative and quantitative methods are used in this research. The probability method (random sampling method) and non-probability method (purposive sampling method) were used in this study.

### Study Area

The study area for this research was the undergraduate students from 10 arts and science colleges University, China. A Simple random sampling method was used for selecting the sample size.

### Data Collection

Both primary and secondary data were collected for this study. The primary data was collected through structured questionnaires and interviews. Secondary data sources were collected from reports, reputable journals, different articles, and standard books. The main tool for primary data collection is structured questionnaires because it gains reliable information.

#### Questionnaire Data Collection

In primary data, a questionnaire survey is the main tool for collecting information. A uniform self-structured questionnaire was designed and questions are based on the research topic. Five-point Likert scale-based questionnaires are developed. Each statements respondent information are measured by using a five-point Likert-scale method, for which 1 = “strongly agree”, 2 = “agree”, 3 = “neutral”, 4 = “disagree” 5 = “strongly disagree.”

#### Interview (Face-to-Face)

In this research, we conduct the semi-structured interview for getting information from people particularly getting knowledge about the topic. The semi-structured interview is typically conducted in a face-to-face situation, allowing the researcher to get fresh insights, ask questions, and evaluate phenomena from many angles.

### Population and Sample Size

The study population consisted of students from 10 arts and science colleges. The sample of data was collected from undergraduate students. A total of 349 sample sizes of college students responded to the questionnaire survey and 12 sample sizes of college teaching staff responded to the face-to-face interview from the priority areas of the university. The gathered data pointed out that only 349 respondents' questionnaires were used for the data analysis after canceling some uncompleted questionnaires. Simple random and purposive sampling methods are used for classifying the data, and 130 from the art department, 90 from the science departments, 69 from the management department, and 60 from the commerce department responded. For the data analysis, the 410 population was believed to be adequate and representative, but we take only 349 respondent questionnaires.

### Participants

**Figure 3:**
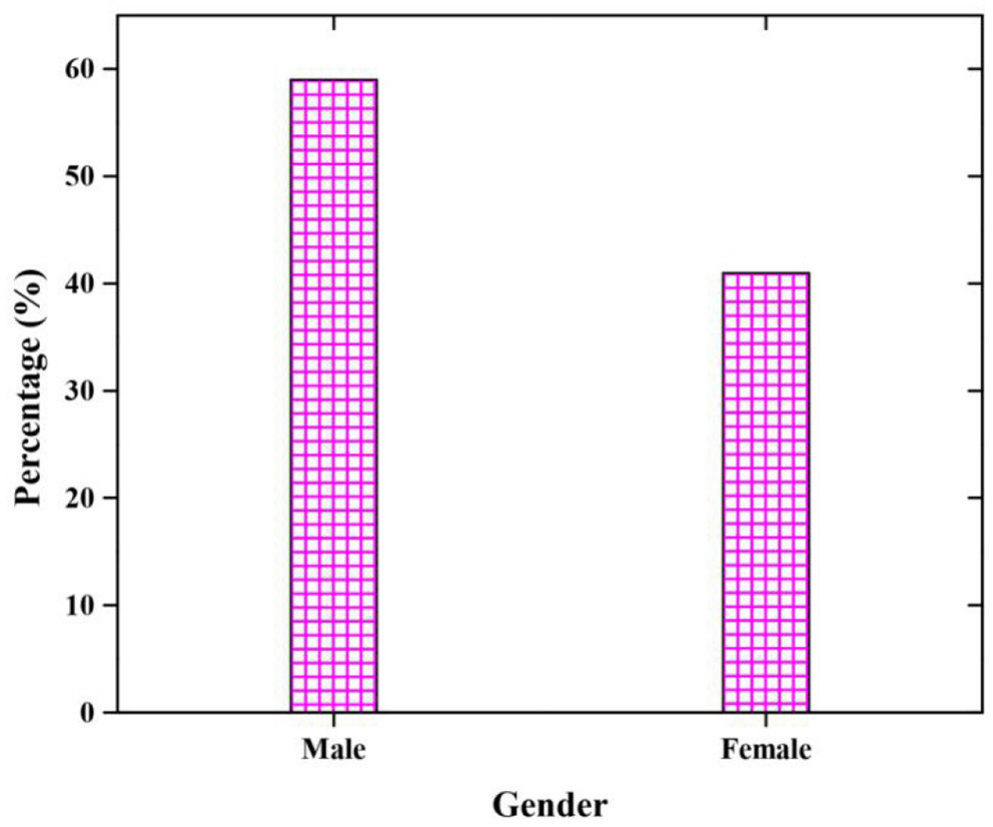
Gender of the respondents.

**Figure 4:**
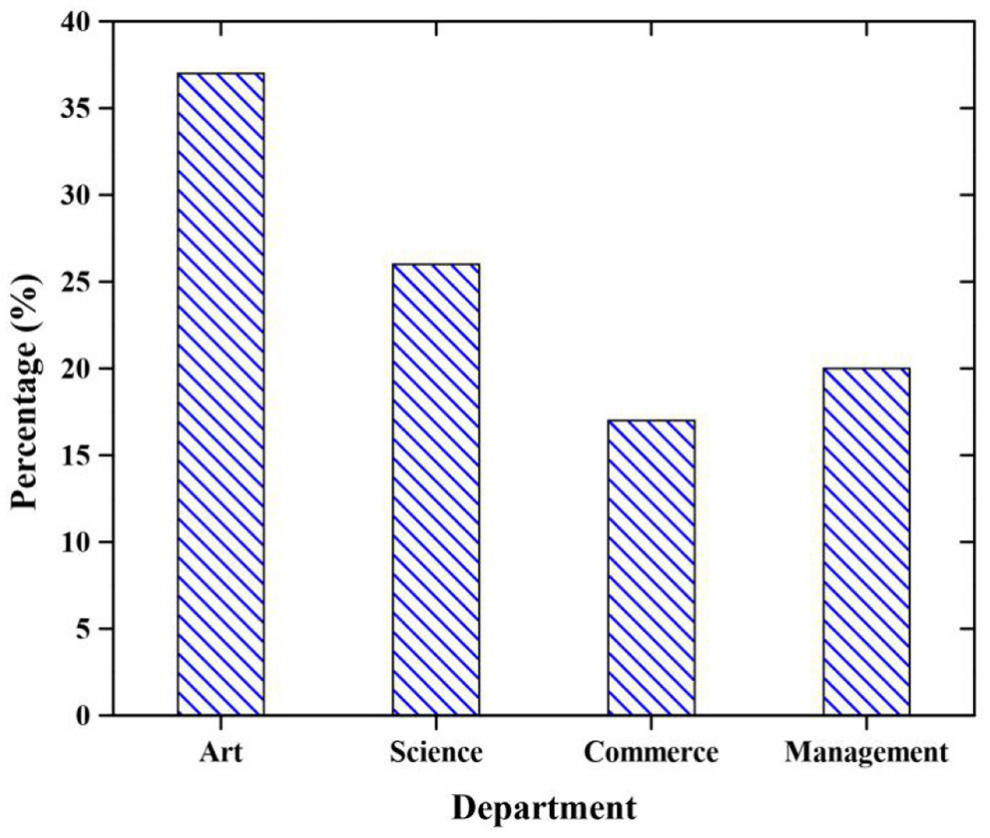
Department of the students.

A total of 410 questionnaires were distributed for a survey, but only 349 respondents' questionnaires are used for the analysis. Table 1 shows that the gender of the students is inferred that 59% of respondents are male and 41% of respondents are female (see Figure 3). Department of the students is inferred that 37% of respondents from the art department, 26% of respondents from the science department,17% of respondents from commerce department and 20% of respondents from management (included tourism) department (see Figure 4).

**Table 1:** Demographic details of the students.

**Demographic Variables**	**Category**	**Percentage (%)**
Gender	Male	59
	Female	41
Department	Art	37
	Science	26
	Commerce	17
	Management	20

### Data Analysis

The acquired data were analyzed using the SPSS 20 software package. The analysis, which was aided by SPSS software, played an important role in getting valid information. It had helped to data analysis and ensures that the SPSS results were valid. The software compared and analyzed the outcomes of many variables utilized in the analysis of the questionnaire. Excel is also used to create illustrations and calculate some of the analytical solutions.

## Result

### The Reliability Analysis

Cronbach's alpha is a measure of internal consistency, closely related a set of statements are as a group. It is considered to be a measure of scale reliability (41). Most of the time, Cronbach's alpha value is used to determine the reliability of internal consistency. In most research contexts, a reliability coefficient of 0.70 or above is regarded as “acceptable.” Using the Cronbach's alpha test, it was also discovered to be internally consistent. Table 2 shows the Cronbach's alpha values were 0.97 for EA, 0.95 for PIS, 0.92 for SA, 0.87 for EM, 0.85 for CN and 0.78 for (PD). The internal consistency of the six key factors indicates that their dependability for this study is acceptable.

**Table 2:** Reliability analysis.

**S/n**	**Major groups**	**Item number**	**Cronbach alpha**	**Result**
1	Self-Awareness (SA)	SA01 To SA05	0.92	Excellent
2	Extracurricular Activities (EA)	EA01 to EA06	0.97	Excellent
3	Professional and Innovative Skills (PIS)	PIS01 to PIS05	0.95	Excellent
4	Personality Development (PD)	PD01 to PD05	0.78	Acceptable
5	Emotion Management (EM)	EM01 to EM05	0.87	Good
6	Cultural Norms (C)	CN01 to CN07	0.85	Good

### Descriptive Analysis

**Figure 5:**
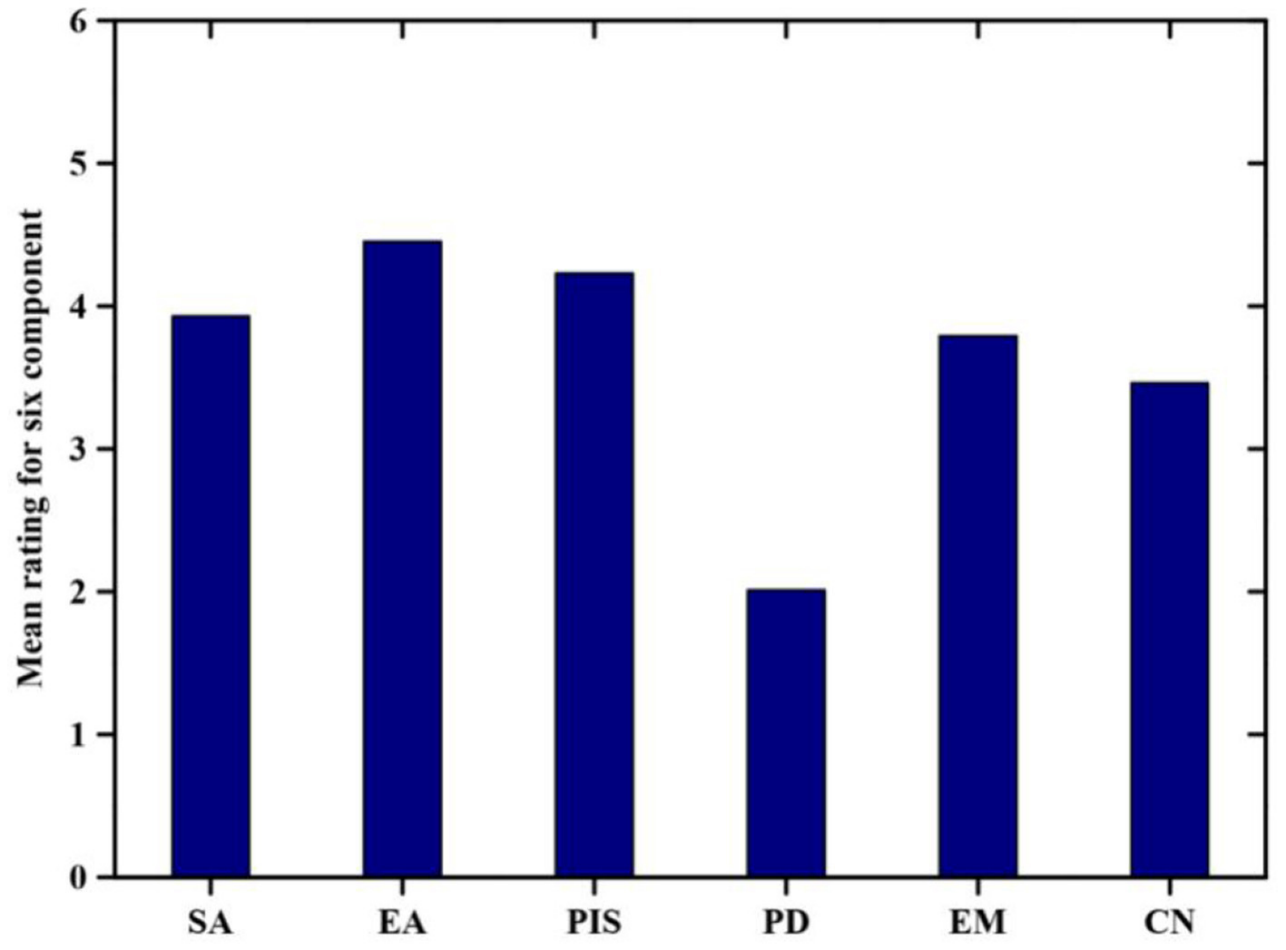
The six distinct factors developed for improving mental health. SA stands for Self-Awareness, EA, Extracurricular Activities; PIS, Personality and innovative skills; PD, Personality Development; EM, Emotion Management; CN, Cultural norms.

Descriptive analysis is one of the important techniques to analyze the data in this study. The analyzed data values are shown in Tables 3, 4. We summed the ratings for all items in each dimension to determine the relative importance of the three generated factors for the students (see Figure 1). In improving students' mental health in education extracurricular activities factor play an important role (*M* = 4.45), Secondly, the professional and innovative skill factors play an important role in improving student mental in education (*M* = 4.23). Thirdly, the self–awareness factor is an important reason offered by students for improving mental health (*M* = 3.93). The fourth emotion management factor significantly plays a role in improving the mental health of students (*M* = 3.79). Fifth, Cultural norms play a significant role in students' mental health (*M* = 3.46). Finally, personality development seems to be a relatively less important factor for improving the student's health in education (*M* = 2.01). The extracurricular activities and professional and innovative skills are the most potential factor offered by students for improving the mental health of students in education (see Figure 5).

**Table 3:** Descriptive statistics analysis.

**Major groups**	**Mean**	**SD**
Self-Awareness (SA)	3.93	0.83
Extracurricular Activities (EA)	4.0.45	0.62
Professional and Innovative Skill (PIS)	4.23	0.79
Personality Development (PD)	2.01	1.20
Emotion Management (EM)	3.79	0.99
Cultural Norms (C)	3.46	1.09

**Table 4:** *T*-test analysis.

**Variables**	**Variables**	**Hypotheses**	***T***	**Df**	**Sig (2-Tailed)**	**Result**
Self- Awareness	Emotion Management	Hypothesis 1	16.367	348	0.000	Supported
Cultural Norms	Self- Awareness	Hypothesis 2	21.081	348	0.000	Supported
Personality Development	Professional and Innovative Skill	Hypothesis 3	17.452	348	0.000	supported
Extracurricular Activities	Personality Development	Hypothesis 4	13.637	348	0.000	supported
Extracurricular Activities	Professional and Innovative Skill	Hypothesis 5	15.335	348	0.000	supported
Self-Awareness	Cultural Norms	Hypothesis 6	19.354	348	0.000	supported

### *T*-Test

**Figure 6:**
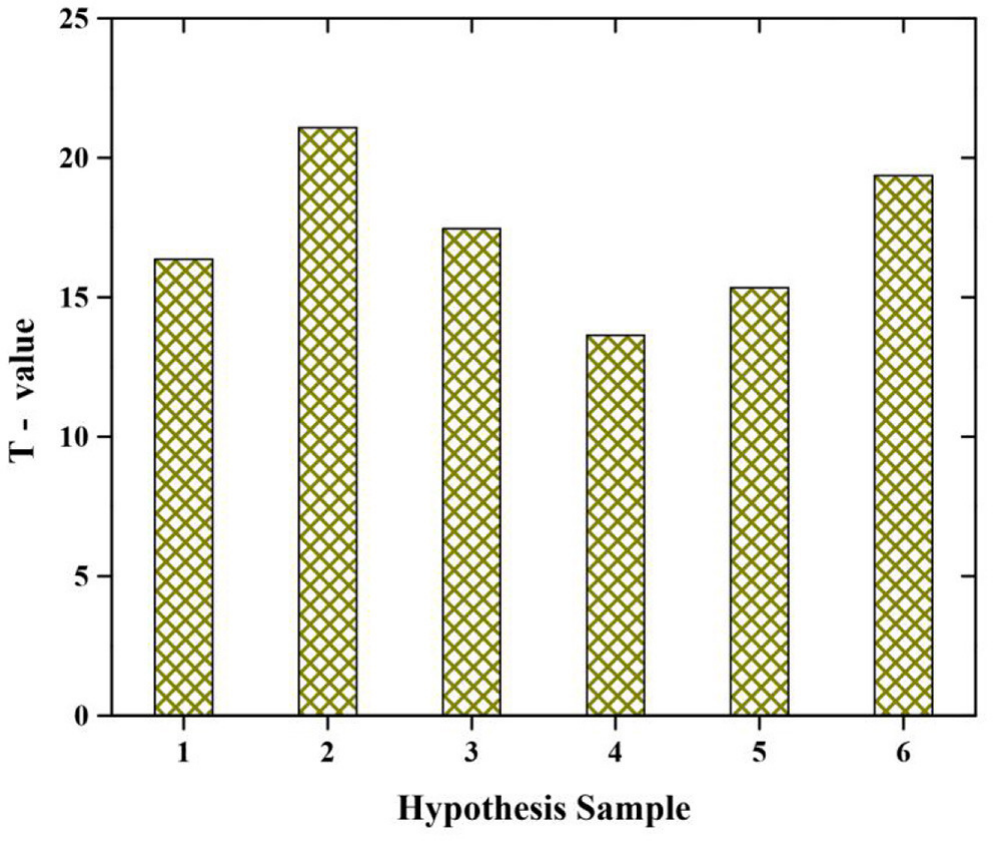
*T*-test hypothesis.

Table 4 shows the result of the hypothesis by using *t*-test. Since the *p-*value is <0.05 the hypothesis is accepted at the 5% level of significance. The first hypothesis, the degree of freedom df is 348 with a *p-*value that is sig is 0.000, denotes there is a significant relationship between self-awareness and emotion management. The second hypothesis, degree of freedom df is 348 with a *p-*value that is sig is 0.000, which means there is a significant relationship between cultural norms and self-awareness. The third hypothesis degree of freedom df is 348 with a *p-*value that is sig is 0.000, it denotes there is a significant relationship between personality development and professional and innovative skill. The fourth hypothesis degree of freedom df is 348 with a *p-*value that is sig is 0.000, which means there is a significant relationship between extracurricular activities and personality development. The fifth hypothesis degree of freedom df is 348 with a *p-*value that is sig is 0.000, it means there is a significant relationship between extracurricular activities and professional and innovative skill. The sixth hypothesis degree of freedom df is 348 with a *p-*value that is sig is 0.000, it means there is a significant relationship between self-awareness and cultural norms. Finally, all the hypotheses are accepted in the analysis (see Figure 6).

## Discussion

Mental health unification can be progressive when the goal of mental health is to contain powerful schooling and the goal of effective schools is to have students' active functioning. Before mental illness develops, little alteration happens in natural thoughts. The dynamic evaluation system was invented by Dimitropoulos. The dynamic evaluation system is a necessary part of the evaluation method and it is not utilized in alignment with this. The necessity of dynamic evaluation is that it investigates to protect victory method evaluation when starting it. The achievement of this article is, with the help of a dynamic evaluation system, the instructor can maintain the mental health of college and university students. We expressed about Canadian mental health policies, participatory model, components of the mental health curriculum, music therapy curriculum system, traditional teaching method, Modern teaching method, and how to maintain faultless mental health in this article. We find six factors for the dynamic evaluation system to protect students' mental health. The factors are extracurricular activities, professional and innovative skills, self-awareness, emotional management, cultural norms, and personality development. If the curriculum is framed by these factors easily students can avoid mental health problems. Extracurricular activities help to learn additional things beyond the subjects. Extracurricular activities modify the students as active personalities. Thinking skills take major sessions in professional and innovative skills. It is being optimism to mental health. Through these skills, students can express their thoughts and ideas with others. So students can maintain good mental health. Understanding our feelings is considered self-awareness. It helps to identify one's feelings and thoughts. It manages the control of feelings. It supports finding the good and uniqueness of a particular person. Emotional management accepts and controls the feelings of the students. Virtue, ethics, honesty, loyalty, responsibility, positive thoughts, relational skills are the important factors in cultural norms. These characteristics present the support to the students to lead the good mental health to the students. Feelings and ways of behavior affect mental health in personality development. Self-confidence is the main element in personality development. We have collected samples from 10 arts and science colleges, especially undergraduate students. We got 349 respondents with the help of a questionnaire survey. Twelve college instructors responded to face to face of the university. Simple random sampling and purposive sampling methods are utilized for this research. The data collected represented that only 349 populations were utilized for analysis, after ignoring a few data that had greater values in the students' answers. We got 130 respondents from arts, 90 from science, 69 from management, 60 from the commerce department. In this analysis, 410 population was enough and typical. Cronbach's alpha test was also utilized for this research, especially in reliability analysis, which was invented to be internally consistent. At last, extracurricular activities and professional and innovative skills are the most valuable factor expressed by students to maintaining the perfect mental health in education.

## Conclusion

We conclude that the support of a dynamic evaluation system protects the mental health of college students. The purpose of the research is extracurricular activities, professional and innovative skills, self-awareness, emotional management, cultural norms, and personality development are the valued factors in a dynamic evaluation system for students' mental health. With the support of 10 arts and science colleges (undergraduate students), we have 349 respondents. A questionnaire survey, Simple random sampling, purposive sampling methods, and Cronbach's alpha test (reliability analysis) were take-up for this research. The information gathered that only 349 students were used for analysis, after avoiding little data that had larger values in the students' answers. We got 130 respondents from various departments like art, science, management, and commerce. Four hundred and ten participants were enough and representative. The result of the article is, in first, an extracurricular activity shows a major part to develop students' mental health. Second, professional and innovative skills lead a part to maintain students' mental health. Third, self-awareness is being an important factor to secure a student's mental ability. Fourth, the Emotion management factor takes a necessity to save a student's mental health. Fifth, cultural norms play a role in students' mental health. At last, personality development takes place in students' mental health. Thus, in this article, extracurricular activities and professional and innovative skills are influential factors when comparing other factors. These factors are articulated by students to maintain their mental health of the students.

## Data Availability Statement

The original contributions presented in the study are included in the article/supplementary material, further inquiries can be directed to the corresponding author.

## Ethics Statement

Ethics approval and written informed consent were not required for this study in accordance with national guidelines and local legislation.

## Author Contributions

The author confirms being the sole contributor of this work and has approved it for publication.

## Conflict of Interest

The author declares that the research was conducted in the absence of any commercial or financial relationships that could be construed as a potential conflict of interest.

## Publisher's Note

All claims expressed in this article are solely those of the authors and do not necessarily represent those of their affiliated organizations, or those of the publisher, the editors and the reviewers. Any product that may be evaluated in this article, or claim that may be made by its manufacturer, is not guaranteed or endorsed by the publisher.
